# Histological and Physiological Effects of Treatment of *Rudbeckia hirta* with Gamma Radiation

**DOI:** 10.3390/plants12122245

**Published:** 2023-06-08

**Authors:** Szilvia Kisvarga, Dóra Hamar-Farkas, Katalin Horotán, Ádám Solti, Edina Simon, Máté Ördögh, András Neményi, Gábor Boronkay, László Orlóci

**Affiliations:** 1 Ornamental Plant and Green System Management Research Group, Institute of Landscape Architecture, Urban Planning and Garden Art, Hungarian University of Agriculture and Life Sciences (MATE), 1223 Budapest, Hungary; kisvarga.szilvia@uni-mate.hu (S.K.); nemenyi.andras.bela@uni-mate.hu (A.N.); boronkay.gabor@uni-mate.hu (G.B.); orloci.laszlo@uni-mate.hu (L.O.); 2 Department of Floriculture and Dendrology, Institute of Landscape Architecture, Urban Planning and Garden Art, Hungarian University of Agriculture and Life Sciences (MATE), 1223 Budapest, Hungary; ordogh.mate@uni-mate.hu; 3 Zoological Department, Institute of Biology, Eszterházy Károly Catholic University, 3300 Eger, Hungary; horotan.katalin@uni-eszterhazy.hu; 4 Department of Plant Physiology and Molecular Plant Biology, Eötvös Loránd University, 1117 Budapest, Hungary; adam.solti@ttk.elte.hu; 5 Anthropocene Ecology Research Group, Eötvös Loránd Research Network, University of Debrecen, 4032 Debrecen, Hungary; simon.edina@science.unideb.hu; 6 Department of Ecology, University of Debrecen, Egyetem Square 1., 4032 Debrecen, Hungary

**Keywords:** *Rudbeckia hirta*, ornamental, urban green, gamma, breeding, annual, gardening

## Abstract

The breeding of resistant, high-yield, decorative ornamental plant varieties may be impacted by climate change in the future. The use of radiation induces mutations in plants, thereby increasing the genetic variability of plant species. *Rudbeckia hirta* has long been a very popular species in urban green space management. The goal is to examine whether gamma mutation breeding can be applied to the breeding stock. Specifically, differences were measured between the M1 and M2 generations, as well as the effect of different radiation doses belonging to the same generation. Morphological measurements showed that gamma radiation has an effect on the measured parameters in several cases (larger crop size, faster development, larger number of trichomes). Physiological measurements (examination of chlorophyll and carotenoid content, POD activity, and APTI) also showed a beneficial effect of radiation, especially at higher doses (30 Gy), for both tested generations. The treatment was also effective in the case of 45 Gy, but this radiation dose resulted in lower physiological data. The measurements show that gamma radiation has an effect on the *Rudbeckia hirta* strain and may play a role in breeding in the future.

## 1. Introduction

Due to the development of new growing areas, where traditional varieties cannot be adapted, the need to produce new varieties of ornamental plants with improved properties has increased [1]. At the beginning of the 20th century, plant biologists established that the frequency and efficiency of genetic modifications in treated seeds could be increased by using chemical and radiation technology [2]. Subsequently, various mutagens, such as physical or chemical mutagens, were used to induce a wide range of genetic variability that led to and contributed to current plant breeding [3]. Radiation-induced mutation breeding is thus a remarkable method that can produce superior mutant varieties, in contrast to traditional breeding such as selection and crossing, which is time consuming and labor intensive with limited genetic trait changes [4,5,6].

Mutation breeding, including natural and artificial mutations, is an excellent method in the field of ornamental plant breeding because many species can be easily propagated, which facilitates the cultivation of spontaneous and induced mutants. The breeding of mutant varieties with the help of an ion beam has already been attempted on many ornamental plants, and some species have also been used to investigate the mutagenesis process. In addition, progress is being made in clarifying the genetic mechanism of the expression of important traits, which will probably result in the development of more efficient mutation breeding methods in ornamental plant breeding [7]. There are several literature references regarding this. In the case of gamma radiation applied to chrysanthemum plants, doses of 10 Gy and 20 Gy resulted in a change in flower color. It was found that gamma radiation affected leaf length, leaf width, stem diameter, stem length, and inflorescence diameter. In addition to morphological changes, it also resulted in histological changes, as changes also occurred in the structure of the leaves [8]. Changes in the morphological characters of *Dendrobium odoardi* Kraenzl. were also observed in individuals [9]. *Tulipa* sp. (L.) gamma radiation (5 Gy) stimulated the sprouting of the bulbs, and the survival rate of the bulbs was also higher. Too-high doses (20–100 Gy) inhibited growth. As the gamma radiation increased, the anthocyanin and flavonoid content decreased. The morphological properties of leaf stomata also changed [10]. In another bulbous ornamental plant, *Narcissus tazetta* (L.) var. *chinensis*, it was observed that gamma radiation given at a low dose (10 Gy) results in a change in growth, while gamma radiation applied at a higher dose brings about other morphological changes in the plants [11]. In the ornamental version of *Capsicum*, gamma radiation caused larger flowers, male sterility, and changed fruit color in the second generation [12]. When the rhizomes of *Cyperus alternifolius* L. were treated with gamma radiation, the lowest (20 Gy) and the highest (100 Gy) dose increased the germination capacity, while the doses between the two caused distorted growth. However, the distortions disappeared in the M2 generation [13]. At the same time, it can also be used to reduce seed germination. In the case of *Pennisetum alopecuroides* (L.) Spreng, the 60 Gy dose reduced the seed yield [14]. Gamma radiation does not always bring the expected result. In the case of *Camellia sinensis* (L.) Kuntze, lower radiation doses increased the germination power, but the seedlings died en masse at the age of five months. When gamma radiation was applied at a higher dose, the germination capacity decreased, but the morphological characteristics became more favorable (larger leaf size, larger flower size, higher height) [15], as in *Adenium obesum* (Forssk.) Roem. and Schult. [16]. In the case of *Lilium*, the number of leaves and the content of chlorophyll changed as a result of gamma radiation [17]. In the case of *Echinacea purpurea* (L.) Moench, the flower color changed and the size of the inflorescence increased, and the shape of the inflorescence and the height of the plant were altered as a result of gamma rays [18]. When *Philodendron erubescens* (K. Koch and Augustin) ‘Gold’ plants were treated with gamma radiation, the plant size, the number of branches, and the color of the leaves changed, which could be the basis for the breeding of a new variety [19].

Tests show that low doses of gamma radiation improve the morphological and biochemical properties of some plants. Gamma ray treatments carried out in the early stages of seed germination promote the synthesis of RNA and protein, thus increasing the growth of seedlings as well as increasing the antioxidant capacity of cells, helping cells to fight against daily stress [20]. Environmental factors can be broadly divided into biotic and abiotic components [21]. Biotic stress is one of the most common causes of the death of ornamental plants, which can be changed by modifying the genetic codes [22]. Biotic factors caused by pests, bacteria, viruses, fungi, nematodes, etc., represent a serious threat to the growth and development of plants, thereby adversely affecting quality. The breeding of stress-resistant plant varieties is becoming very important in the current agricultural system. Mutagenesis is one of the most common techniques for controlling plant stress. To induce mutagenesis, plant breeders use technologies such as physical (gamma radiation, ultraviolet radiation, etc.), chemical (ethyl methanesulfonate, methyl methanesulfonate, sodium azide, etc.) and genetic (ZFN, TALEN, and CRISPR) technologies [21]. These technologies are also increasingly used in ornamental plant breeding. *Osmathus fragrans* Lour. is very sensitive to salt stress. However, with gamma radiation, salt tolerance can be achieved in the species, which becomes more and more noticeable as the dose increases. In addition, it moderated the MDA (melonaldide) level, which was associated with a significant increase in superoxide dismutase (SOD), peroxidase (POD), and catalase (CAT) activity. The accumulation of proline can also contribute to increasing the tolerance against salt stress [23]. In the case of *Paeonia* x *suffruticosa* Andrews, it was established that 30 and 40 Gy gamma radiation affected the physiological and biochemical state of the plants. As a result of the treatment, the activity of antioxidant enzymes, including superoxide dismutase, peroxidase, and catalase, gradually increased up to the value of 40 Gy. Total soluble protein content progressively decreased, while proline and malondialdehyde content increased significantly [24]. An increase in salt stress resistance due to gamma radiation can also be observed in *Musa* L. individuals under in vitro conditions [25]. Gamma radiation improved powdery mildew resistance in *Gerbera jamesonii* ‘Harley’ [26]. Gamma radiation can also change the histological properties. *Catharanthus roseus* (L.) G. Don. gamma radiation had an effect on the callus biomass growth of individuals, and it also had an impact on the vincristine and vinblastine content under in vitro conditions. Callus growth was maximal at 20 Gy but decreased at 100 Gy [27]. In the case of *Dimocarpus longan* J. de Lour., gamma radiation modified the morphological features and the rate of photosynthesis [28].

### Rudbeckia Hirta as a Genetic Resource

The genus *Rudbeckia* consists of approximately 30 species native to North America [29]. The genus includes annual, biennial, and perennial species [30]. The annual species *Rudbeckia hirta* includes diploid (2n = 2x = 38) and tetraploid (2n = 4x = 76) cultivars with varied flower color and flower shape, typically flowering in late summer and autumn. *Rudbeckia* is a popular ornamental taxon, suitable for urban settings due to its ornamental diversity, low maintenance, and heat and drought tolerance [31]. In Hungary, Dr. Zoltán Kováts dealt with the breeding of the species [32].

*Rudbeckia*s are highly adaptable species with valuable flowering as ornamental plants. Complementing the current varieties with new cultivars would be important in trade. Interspecific hybridization and induced polyploidy could be a significant improvement of the genus from an ornamental horticultural point of view. When comparing diploid and tetraploid individuals, induced polyploidy significantly reduced overwintering ability [33]. Due to the aesthetic value of perennial herbaceous flowering plants, such as colorful flowers, more attention is paid to these plant groups in the field of urban planting [34].

*Rudbeckia hirta* is a very popular species worldwide, several Hungarian varieties of which are used in public areas. ‘Mackó’, ‘Aranyálom’, ‘Kokárdás’, ‘Sárgarigó’, and ‘Őszifény’ are Hungarian cultivars found in many cities. These products were bred by Dr. Zoltán Kováts in the 1980s and 1990s at the Horticultural Research Institute, which today operates as part of MATE. The climate at that time was not characterized by the summer drought typical of current years, and heat waves lasting several weeks, as well as many diseases, were not present in those decades. These breeds can no longer cope with the current environmental problems. Another important factor is that the market is looking for compact, resistant varieties with many flowers and special flower colors or flower shapes. The old *Rudbeckia hirta* varieties cannot satisfy this demand. Although the classic crossbreeding and selection breeding methods are effective, producing a new variety using these methods is costly and time consuming. Mutational breeding, on the other hand, can lay a new foundation for the faster and more cost-effective production of climate-tolerant and market-demanded varieties. Treating strains of *Rudbeckia hirta* ‘Őszifény’ produced by selective breeding with gamma rays can be a suitable method to increase genetic variability.

After several years of selective breeding of the *Rudbeckia hirta* individuals used, they were treated with a dose of gamma radiation, and the M1 and M2 generations were also examined. We studied the effect of the applied radiation doses on the given breed. As a result of the treatments, we aimed to determine a dose or doses with which we can continue to work in the future. When determining this, it is important to compare the morphological and physiological results. Another goal of ours was to select the groups and doses of the cultivated plants with which we can pursue the breeding program and make them resistant to the current climate (abiotic and biotic stress effects). Our goal is to examine whether gamma mutation breeding can be used for the applied breed candidate, and if so, the question of how this process takes place becomes important.

During our studies, we specifically measured the differences between the M1 and M2 generations as well as the effect of different radiation doses belonging to the same generation on the morphological and physiological properties. Recognizing the difference between generations, the goal was to measure the changes in the characteristics of *Rudbeckia hirta* varieties produced through sexual intercourse between generations. This can be an important clue in subsequent gene conservation and breeding tasks.

## 2. Results

### 2.1. Morphological Evaluation

#### 2.1.1. Leaf Cross-Section

**Figure 1:**
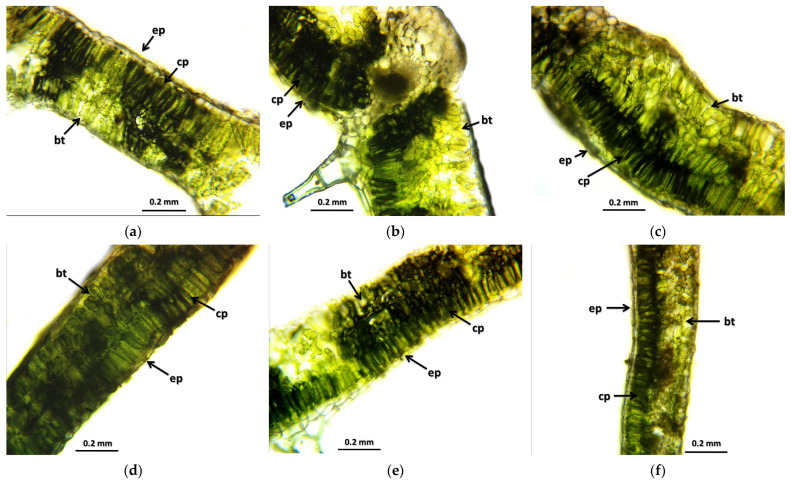
Leaf cross-samples of *Rudbeckia hirta* with gamma radiation treatment: (**a**) control; (**b**) 5 Gy M2; (**c**) 10 Gy M2; (**d**) 30 Gy M2; (**e**) 30 Gy M1; (**f**) 45 Gy M1. The abbreviations shown in the pictures mean the following: ep—epidermis; cp—columnar parenchyma; bt—basal tissue.

Regarding the results of the morphological evaluation, it can be said that the radiation had an effect on both the leaf and stem cross-sections. Sampling took place in full bloom, which in Hungary happens at the beginning of September—by this time, in the case of several individuals, the foliage is already starting to age. This is clearly visible in Figure 1, where, although the cells of the epidermis are closely aligned and the basal tissue cells are intact, the spongy parenchyma breaks, and chlorophyll-deficient spots begin to form.

This is also evident in the leaf cross-section photos of the 5 Gy M2 stock (Figure 1b); the cells under the epidermis have disappeared, but the existing cell rows are still more closely aligned than in the case of the control group. In the case of the M1 group that received a stronger concentration, of 30 Gy M1 (Figure 1e), it can be said that this group shows the most stable tissue organization among the M1 populations. Its cells are uniform, their cell walls are closed, and no vacuoles have formed. The spongy and columnar parenchyma of the base tissue is uniform and rich in chlorophyll. In the 30 Gy M2 group (Figure 1d), the effect of fresh irradiation is visible: the leaf tissue diameter is irregular, the cells of the epidermis are thin, the basal tissue is damaged in some places but is still more complex than the leaf diameter of the control group. In the case of 45 Gy M1 (Figure 1f), it appears that this dose is probably close to the maximum usable level: the cells are smaller, the cell wall is strong, and the cell row of the epidermis is ordered. The cells of the epidermis and the columnar parenchyma are arranged and, no vulvae and cell death can be observed.

#### 2.1.2. Stem Cross-section

**Figure 2:**
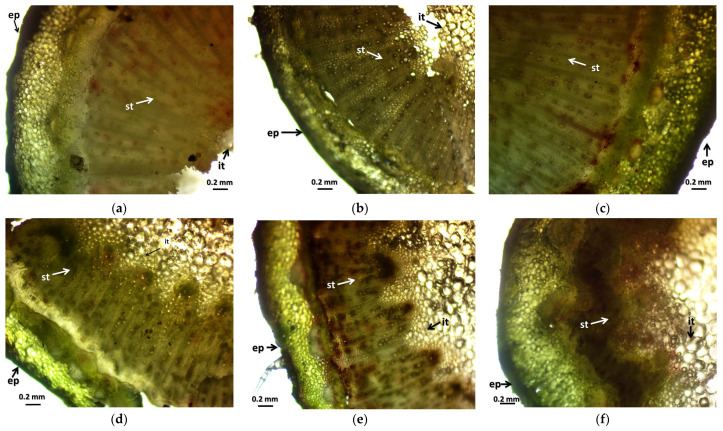
Stem cross-samples of *Rudbeckia hirta* with gamma radiation treatment: (**a**) control; (**b**) 5 Gy M2; (**c**) 10 Gy M2; (**d**) 30 Gy M2; (**e**) 30 Gy M1; (**f**) 45 Gy M1. The abbreviations shown in the pictures mean the following: ep—epidermis; st—secondary thickening; it—internal tissue.

Looking at the experiences of the stem cross-section, the results seen in the leaf cross-sections are also visible. The effect of gamma radiation is evident in all treated groups. In the case of the control group (Figure 2a), it can be observed that the degree of secondary thickening is not considerable. 

Although the epidermis is strong, the cell row under the epidermis is thick, and the cells are intact, the cells of the intestinal tissue do not form well-developed, distinguishable tissue groups, and the central part of the intestinal tract is missing. In the case of M2 masses (Figure 2b–d), the central part of the intestinal tissue also fades, but the degree of secondary thickening is much stronger, and this is directly proportional to the increase in radiation dose. This effect can be observed even more intensely in the M1 stocks, also in direct proportion to the increase in radiation dose. Here, the cells of the central intestinal tissue are intact, most of the cells have a strong cell wall, the cells of the epidermis are strong, and some tissues and cell groups of the basal tissue are distinctly separated from each other. Furthermore, the dermis is thickened.

#### 2.1.3. Trichomes

**Figure 3:**
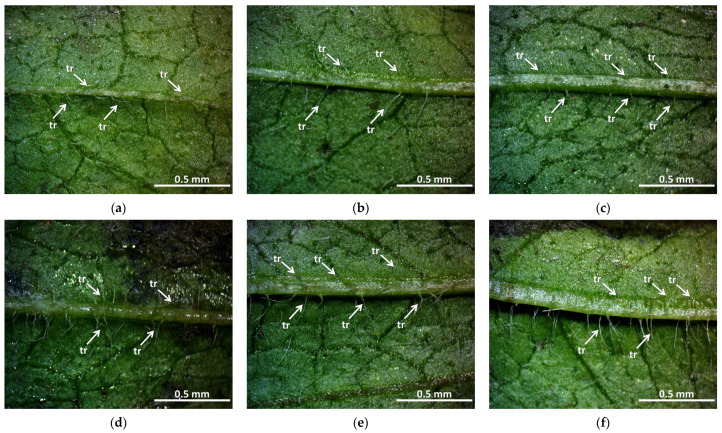
Trichomes of *Rudbeckia hirta* with gamma radiation treatment: (**a**) control; (**b**) 5 Gy M2; (**c**) 10 Gy M2; (**d**) 30 Gy M2; (**e**) 30 Gy M1; (**f**) 45 Gy M1. In the case of M2 stocks, the number of trichomes decreased (5 Gy M2, Figure 3b) or increased (10 Gy M2 and 30 Gy M2, Figure 3c,d). In M1 stocks, their number increased (30 Gy M1, 45 Gy M1, Figure 3e,f) and their length became longer (45 Gy M1). The marked difference between the M2 and M1 stocks is outstanding—in the 30 Gy M2 group, the trichomes are much more uniform and stronger than in the case of M1. The number of trichomes were counted using visual observation. The abbreviation shown in the pictures means the following: tr—trichomes.

The arrangement and number of trichomes can also be an important morphological sign in terms of observing the effects of gamma radiation treatment. As can be seen in the photos, we also found differences in the size and number of trichomes. While in the control group (Figure 3a) the trichomes on the main veins of the leaf blades are uniform in size but weak, this is apparently different in the treated stands. 

#### 2.1.4. Crop Length

**Figure 4:**
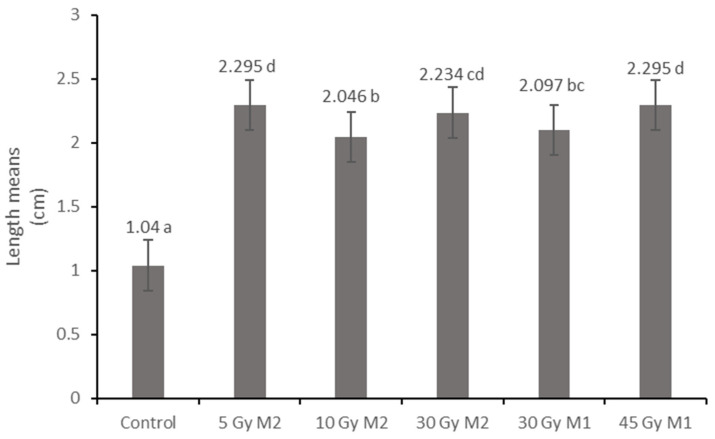
Crop length of *Rudbeckia hirta* with gamma radiation treatment. Different letters indicate significantly different groups (Tukey, *p* > 0.05). *p* = 0.000.

The effect of gamma radiation can also be seen in the measurement results of crop length. In the case of the control group (Figure 4), the average seed length was 1.04 cm. 

**Figure 5:**
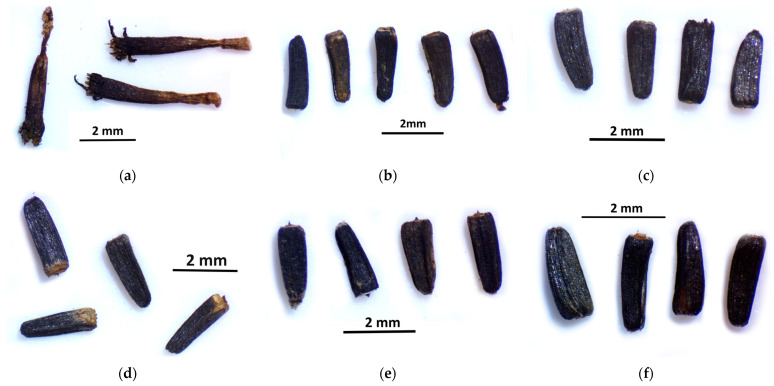
Crop length of *Rudbeckia hirta* with gamma radiation treatment: (**a**) control; (**b**) 5 Gy M2; (**c**) 10 Gy M2; (**d**) 30 Gy M2; (**e**) 30 Gy M1; (**f**) 45 Gy M1.

In addition, the control group contained several leeched seeds (mostly empty seed shells were typical, as in Figure 5a), though in the case of the groups that received gamma radiation, the fruits were healthy and uniform. 

Significant differences were also observed in several cases in the treated groups. The 5Gy M2 group (Figure 5b) had the highest average crop length (2.295 mm) and the 10 Gy M2 group (Figure 5c) had the lowest (2.046 mm).

### 2.2. Physiological Results

Physiological measurements can also reveal a lot about the treated groups. The chlorophyll and carotenoid contents presented below, as well as the peroxidase enzyme activity results, were supplemented with a proline measurement, but this did not produce statistically verifiable results, so the results of this are not presented.

#### 2.2.1. Chlorophyll and Carotenoid Content

**Figure 6:**
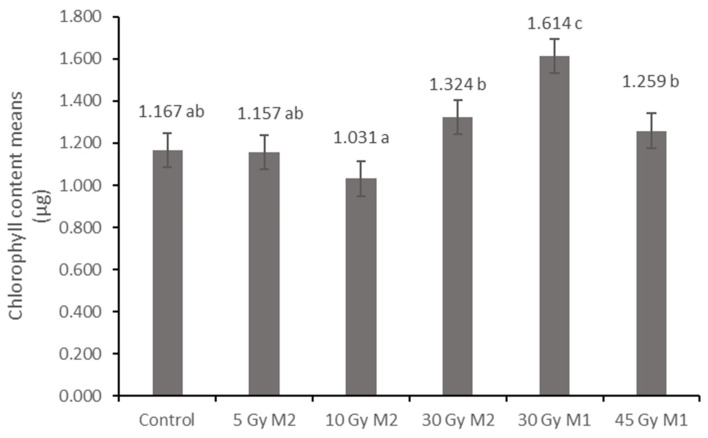
Average chlorophyll content of *Rudbeckia hirta* with gamma radiation treatment. Different letters indicate significantly different groups (Tukey, *p* > 0.05). *p* = 0.000.

The chlorophyll content results show significant differences in several cases. The average chlorophyll content of the 10 Gy M2 group was the lowest (1.031 µg), while the 30 Gy M1 group had the highest value (1.614 µg). The chlorophyll levels of the 30 Gy M2 (1.324 µg) and 45 Gy M1 (1.259 µg) groups show a statistical difference from these (Figure 6).

**Figure 7:**
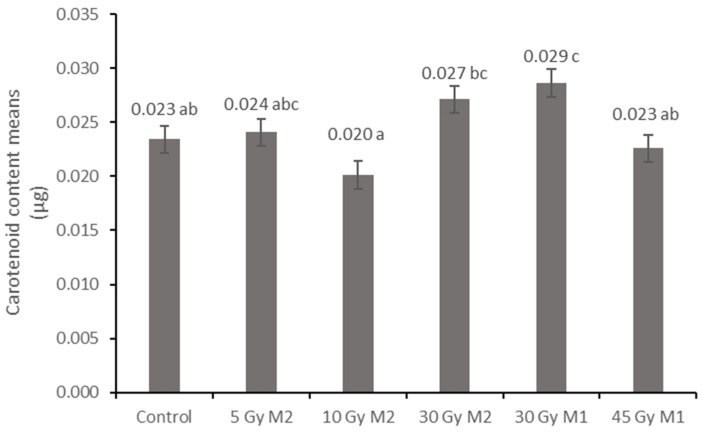
Average carotenoid content of *Rudbeckia hirta* with gamma radiation treatment. Different letters indicate significantly different groups (Tukey, *p* > 0.05). *p* = 0.001.

Similar tendencies of the carotenoid contents were observed, as in the case of the chlorophyll levels. The lowest carotenoid content (0.020 µg) was measured in the 10 Gy M2 group, which is statistically different from the results of the other measurement groups. The highest carotenoid level was detected in the 30 Gy M1 group (0.029 µg). The other groups ranged between the two values (Figure 7).

#### 2.2.2. POD Activity

**Figure 8:**
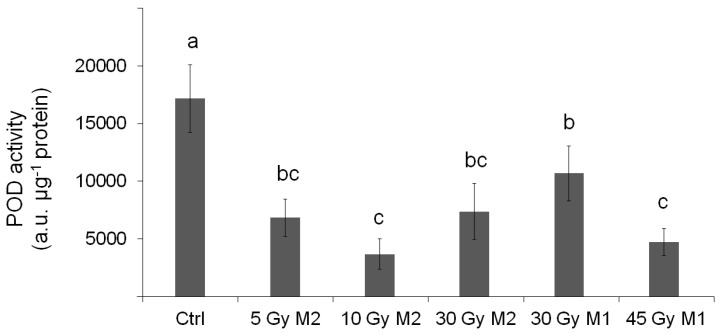
Total POD activity in *Rudbeckia hirta* samples. Error bars indicate SD values. To compare differences in the total POD activity, a one-way ANOVA with a Tukey–Kramer post hoc test was performed (*p* < 0.05). Letters indicate statistical groups.

Compared with the control, the total POD activity decreased in all samples (differences are significant, *p* < 0.05). Among the treated samples, 30 Gy M1 showed the highest total POD activity, but even in this sample the POD activity suffered more than one-third drawback in the activity (to 62.2 ± 14.0% of the Ctrl). In comparison, the total POD activity in 10 Gy M2 and 45 Gy M1 was only 21.5 ± 7.7% and 27.5 ± 6.9% of the control, respectively, indicating a significant effect of the applied treatments on the overall POD spectrum (Figure 8). 

#### 2.2.3. Air Pollution Tolerance Index

Although the air pollution tolerance index (APTI) values of both Ganguly and Mukherjee [35] and Singh et al. [36] measured on the scale show no significant difference among groups using one-way ANOVA (F = 1.422, *p* = 0.183), the results can still be presented due to the APTI values (Table 1).

It can be seen that all groups fall into the intermediate category according to all scales; however, it is striking that the M2 value of the control group and the 5 Gy M2 group is 18, while the M2 value of 10 Gy is 22, and the M2 value of 30 Gy is 20. The APTI values of the M1 generation are 17 and 18. It is clear that urban stress tolerance increased in the groups that received a higher dose of M2. Among the parameters of APTI, there were significant differences based on the relative water content (RWC) (F = 2.681, *p* = 0.006), ascorbic acid content (AAC) (F = 4.095, *p* < 0.001), and pH level (F = 18.028, *p* < 0.001); however, in the case of chlorophyll concentration, significant differences were not found among groups (F = 1.075, *p* = 0.412).

## 3. Discussion

In the course of our work, we dealt with the gamma irradiation treatment of *Rudbeckia hirta* and determined if the breeding stock strain we created is suitable for this type of treatment. If so, the breeding process can be facilitated by this. The effects of gamma radiation were measured and evaluated using histological and physiological methods. This revealed the effects of gamma radiation on organs, cells, and tissues, as well as the magnitude of the stress level (POD, APTI). Since POD isoforms are diverse both in their location as well as their function, alterations in the isoform composition reflects the stress defense and the cell wall composition properties of the plants [37]. The measurements were carried out with seeds irradiated with different doses, and the measurements of two consecutive generations were also compared.

It was found that the treatment of seeds can be a suitable method in the breeding process, as Oladosu et al. [2] established. A change in phenotypic properties was observed in all of the treated plants, which was also evident in the M2 generation in many cases, in connection with the findings of Beyaz and Yildiz [4].

The results of the leaf and stem cross-sections showed that by increasing the radiation dose, the leaf and the stem retain their tissue youth for a longer time. The central axis of the intestinal tissue of the stem is less or not hollow, the cells of the epidermis and dermis are stronger, and the cell walls are uniform. The degree of secondary thickening in the stem increases as the dose increases. There is also a difference between the M1 and M2 generations. The described characteristics also appeared in the M2 generation in the case of the leaf cross-section, not as strongly as in the case of M1 but perceptibly. This is similar to the findings of Susila et al. [8], Hu et al. [11], and Li et al. [10]. The columnar parenchyma cell groups full of chloroplasts visible in the cross-section of the leaf can also show a very close relationship between the amounts of chlorophyll and carotenoids. According to them, the 10 Gy M2 group had the lowest chlorophyll and carotenoid measurements, while the 30 Gy M1 represented the highest chlorophyll and carotenoid values. This assumes some analogy with the microscopic images of leaf cross-sections. The higher doses of gamma radiation initially affected the chlorophyll and carotenoid content, which are the determining factors for the vitality and good stress tolerance of plants. It should be mentioned in this context that the chlorophyll and carotenoid content measured in the 30 Gy M2 and 45 Gy M1 groups—although it does not show a statistical difference with most of the measured groups—is still high. For the *Rudbeckia* strain, doses of 30 Gy M1 and 45 Gy M1 were found to be more effective than lower doses. These results differ from those of Li et al. [10], in which a dosage strength above 20 had an inhibitory effect on several morphological and physiological processes in *Tulipa* bulbs. This is surprising, because for perennial plants with an overwintering organ the optimal dosage strength is assumed to be higher. Likewise, our results are not similar to the findings of Rêgo et al. [12]. This can also be explained by the density of the trichomes of the groups treated with a higher dose, which play a major role in preserving the plants’ greater vitality.

The amount and length of the trichomes also changed in the main leaf veins of the irradiated groups. This is more visible at higher doses (30 Gy M1 and 45 Gy M1). In terms of generations, it is more even in the M2 generation. In summary, it can be said that the trichomes were stronger in all treated groups than in the control group—this may be related to better drought and climate tolerance, which is a very important breeding goal and aspect. This result is similar to the findings of Bhoi et al. [21], which state that the breeding of stress-resistant plant varieties is becoming crucial in the current agricultural system.

The effect of gamma radiation can also be seen in the size, morphological properties, and physiological state of the fruits. The seeds of the control group were not ripe at the time of sampling, and most of the seeds remained unripe. In the case of the groups that received gamma radiation, the fruits were uniform and healthy. In this regard, the radiation also helped vitality, increasing the survival chances of plants, as Ulukapi et al. [20] also stated.

The physiological results also largely reflect the experiences and conclusions presented so far. It is clear from the results of the peroxidase enzyme activity measurements that the enzyme activity of the samples taken in the climate of the end of summer in Hungary shows significantly higher values—in connection with the findings of Geng et al. [23], we achieved a similar result. The peroxidase enzyme is enriched when the plant is stressed. In the case of treated plants, the level of enzyme activity is significantly lower—this contradicts the findings of Wang et al. [24]. From this, we can conclude that the statistically verifiable lower stress level of plants treated with gamma radiation is due to the effects of gamma radiation. Bhoi et al. [21] also stated that mutagenesis is one of the most common techniques for controlling plant stress. In this case, there was no significant difference between the generations. 

In this context, the APTI level did not show a statistically verifiable difference, but it is worth highlighting that the urban stress tolerance is higher at higher doses of the M2 generation.

In summary, it can be said that *Rudbeckia hirta* is a suitable species for use in urban green spaces. Hungarian-bred varieties no longer correspond to the effects of today’s climate and old varieties are no longer fashionable—this is essential in ornamental plant breeding. Gamma radiation had a detectable and favorable effect on the strain of *Rudbeckia*. Stronger doses showed more favorable phenotypic characteristics. The difference between the generations was also noticeable, and in many cases the positive effects of the treatments were preserved. During our measurements, we came to the conclusion that gamma radiation is a suitable method for the breeding of *Rudbeckia hirta* as it promotes the breeding of new, stress-tolerant varieties. This was confirmed by histological and physiological methods.

In the future, it will be an increasingly important factor to breed varieties for the urban environment that tolerate the changed climate and, at the same time, the urban biotic and abiotic stress effects. The Hungarian breeds that are currently in development form a very important genetic basis, but they no longer meet the challenges of today. Mutational breeding, on the other hand, can mark a new direction and a new way to create varieties from old varieties that can be used in urban public areas of the 21st century. Our current results show that generational changes and higher applied doses are an important step for us in terms of breeding. With these results, we can contribute to the creation of an environmentally conscious, more sustainable urban plant application.

## 4. Materials and Methods

### 4.1. Characteristics of the Selected Rudbeckia

**Figure 9:**
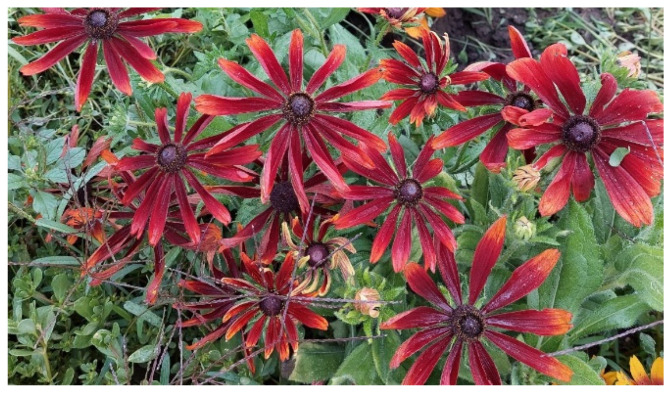
Selection strain no. 5 of *Rudbeckia hirta* ‘Őszifény’.

*Rudbeckia* is similar to the basic variety but its habit is more compact (50–60 cm), it has a larger number of inflorescences, and the burgundy and brown stains are more intense and stronger, often covering the entire petal, so the yellow color does not appear (Figure 9).

### 4.2. Preliminary Treatment

The seeds were treated with gamma rays at the Seibersdorf Plant Breeding and Genetics Laboratory station in 2021 and 2022. Each experiment was repeated 3 times and 30 plants were examined in all groups. The plants were grown in a random block arrangement under outdoor conditions.

The seeds were irradiated at the following doses in the year in parentheses. Thus, the investigated dose effects were examined on M1 or M2 generations. For the M2 generation, plants were grown from irradiated seeds, and we collected the seeds in 2021. They were then cultivated in 2022.

- M2 (2021)—5, 10, 30 Gray
- M1 (2022)—30, 45 Gray.

### 4.3. Making Microscopic Images

The core examinations were performed with a Delta Optical SZ-450T type trinocular stereomicroscope, which has a stepless zoom of 10–45x.

The Euromex bScope BS.1153-PLi microscope was used for the light microscopic examinations.

Leaf cross-section eyepiece: Lens: PLi 10/0.25 Eyepiece: WF120×/20.

Stem cross-section: Lens: PLi 4/0.1.

Leaf cross-sections were cut with a manual sled microtome.

The stem cross-section was obtained by manual pruning with a scalpel.

In both cases, the Levenhuk M1400 plus camera was used to take the microscopic images.

### 4.4. Preparation and Microscopic Examination of Leaf and Stem Cross-Sections

The cut leaf was lifted from the knife into a watch glass filled with water using a soft, wet brush. After cutting the samples belonging to 1 leaf, we lifted the leaf samples onto the slide with a wet, soft brush. Afterwards, we dropped water on it and covered it with a coverslip and examined it under the microscope.

In the case of the treated and control plants, a cross-section was made from several points of the leaf, which was examined and photographed under a light microscope. After that, secondary thickenings and changes were examined. The Topuview program was used to take the photos and the Fiji program was used for the measurements.

The stem was cut manually with a scalpel then placed on a glass slide. Water was dropped on it, it was covered with a coverslip and placed under the microscope to be examined, and photographs were taken.

### 4.5. Stereomicroscopic Examination of Seeds

For each group, 1 inflorescence was collected. In order to not lose seeds, this was collected by pulling the paper bag over the inflorescence, tying it, and then cutting it off the stem. Then, the dropout took place in the bag. The samples collected in this way were sorted under a stereomicroscope so that plant debris did not get between the seeds. After that, the seeds were counted to obtain the total number of seeds, and then the leech seeds and healthy seeds were sorted out, which were also counted. The seeds were examined under the stereomicroscope with 2 types of magnification so that the size and surface differences between the individual groups became clearly visible.

### 4.6. Stereomicroscopic Examination of Leaf Hairiness

In the case of the examined groups, different hairiness was observed on the leaf surface. To quantify this, we used the main vein of the dorsal part of the leaf since the number of trichomes on the leaf surface could not be counted. Based on the image taken from the dorsal part, it was counted manually, marking the individual trichomes. In addition, a recording was made of the hairiness of the leaf edge.

### 4.7. Physiological Assessment

#### 4.7.1. Measurement of Chlorophyll and Carotenoid Content

The measurements were made from the plates of the examined leaves based on the methodology of Helrich [38]. Until the physiological tests, the leaf samples were stored in a plastic, zippered bag in a freezer. For the chlorophyll and carotenoid analysis, 3 × 100 mg leaf samples from each group were measured. These were ground in a pestle and mortar with a small amount of quartz sand, then the crushed material was filled into a measuring cylinder and diluted to 5 mL (the diluting solution consisted of a mixture of 80% acetone and distilled water). After that, the sample was shaken (so that the rubbed substances dissolve sufficiently), then it was placed in a test tube and covered with paraffin for a day. Thus, quartz sand and larger particles that adversely affect the measurement settled to the bottom of the solution. The next day, the supernatant of each sample was placed into a cuvette using a pipette and analyzed using a Genesys 10vis spectrophotometer. The instrument measured the solution at 480, 644, and 663 nanometers. The following equations were used to calculate the total chlorophyll and carotenoid amounts from the results obtained in this way:

chlorophyll (a + b) μg/g = (20.2 × A644 +8.02 × A663) × V/w

carotenoid μg/g = (5.01 × A480)/w

where: V = amount of tissue extract (10 mL); w = mass of tissue (0.1 g); A = absorbance.

#### 4.7.2. Peroxidase Enzyme Measurement

The activity of class-III peroxidase isoforms (POD; EC 1.11.1.7) was measured according to Rao et al. [39] and Solti et al. [40]. Briefly, 500 mg frozen leaf material mixed from multiple individual leaves was homogenized with 1 mL isolating buffer: 50 mM Na-K-phosphate buffer, pH 7.0, 1.0 mM EDTA, and 0.1% (*w*/*v*) Triton X-100 and centrifuged for 20,000× *g* 20 min at 4 °C. Supernatant was solubilized in 5 mM Tris-HCl, pH 6.8, 0.01% (m/V) SDS, 10% (*v*/*v*) glycerol, and 0.001% (m/V) bromophenol blue. Proteins were separated on 10–18% gradient polyacrylamide gels as in Solti et al. [40]. POD activity was developed in 50 mM acetate buffer, pH 4.5, 2 mM benzidine, and 3 mM H_2_O_2_. The enzyme activity was terminated in 50% (*v*/*v*) methanol. After digitization using an Epson Perfection V750 PRO gel scanner, densities were measured using Phoretix v 4.0 (Phoretix International, Newcastle upon Tyne, UK). POD activity was normalized based on the total protein contents, determined as in Sárvári et al. [41].

#### 4.7.3. Calculation of Air Pollution Tolerance Index

The level of air pollution can be expressed by the air pollution tolerance index (APTI) as an indirect reaction of plants. High values of APTI indicate low sensitivity, while tree species of low APTI can be considered biological pollution indicators [42,43,44] (Table 2).

APTI values were calculated based on the ascorbic acid content in mg/g (A), total chlorophyll content in mg/g (T), pH of leaf extract (P), and relative water content (R) of the leaves. Using these parameters, we applied the equation proposed by Singh et al. [36]:

APTI = [A × (T + P) + R]/10

The ascorbic acid content was measured with the redox titration method, where 2 g of leaf tissue was crushed and homogenized with 50 mL of water in 3–4 parts. After that, we collected the extract and made up 100 mL in volumetric flasks. From this extract, the leaf pH was first measured using a digital pH meter. After pH measurement, 20 mL portions of the sample were titrated in triplicate with 0.0025 mol of iodine solution in 1 mL of 0.5% starch solution. The blue color remained for 20 s. Chlorophyll was extracted from approximately 50 mg of fresh leaves using 5 mL of 96% ethanol. The absorbance of the extracts was measured at wavelengths of 653, 666, and 750 nm using spectrophotometric analysis. The total chlorophyll content (T) was calculated as follows:

T mg/g = (17.12 × E666 − 8.68 × E653) × V/m ×1000

where V is the volume (mL) of the leaf extract, m is the fresh weight (g) of the leaf sample, and E666 and E653 are the absorbance levels at 666 nm and 653 nm minus the absorbance level at 750 nm, respectively. For the pH measurement, 2 g of leaf tissue was crushed and homogenized in 100 mL of deionized water. To determine the relative water content, the fresh weight of individual leaves (FW) was measured. Then, the leaves were immersed in water overnight before being weighed again to determine the turgid weight (TW). Finally, the leaves were dried in an oven at 70 °C to measure the dry weight (DW). The relative water content (R) was calculated as follows:

R (%) = (FW − DW)/(TW − DW) × 100

### 4.8. Statistical Evaluation

Microsoft Office 365 Excel was used to document our measurement data, and Microsoft Office 365 Word was used for text editing. The processing, comparison, and examination of measurable differences in our results were carried out with the IBM SPSS Statistics 26 program using the ANOVA method. In order to achieve a normal distribution, a part of the database (fresh root mass, fresh green mass, dry root mass, dry green mass) was transformed and the Winsorization method was applied. In all cases, the measured data were analyzed at a 95% reliability (significance) level. Having evaluated the Levene test, if the Sig. > 0.05 the Tukey’s test was used, and if Sig. < 0.05, the Games–Howell post hoc test was used.

## Figures and Tables

**Table 1:** Urban tolerance of groups of *Rudbeckia hirta* treated with gamma radiation.

	RWC	AAC	pH	Chlorophyll	APTI	Ganguly and Mukherjee [35]	Singh et al. [36]
*Rudbeckia* K	76.868	4.243	8.39	15.121	18	intermediate	intermediate
*Rudbeckia* 5 GY M2	93.326	3.252	8.55	18.738	18	intermediate	intermediate
*Rudbeckia* 10 GY M2	154.185	2.211	8.42	20.543	22	intermediate	intermediate
*Rudbeckia* 30 GY M1	74.374	3.586	8.49	25.769	20	intermediate	intermediate
*Rudbeckia* 30 GY M2	70.563	3.686	8.76	21.560	18	intermediate	intermediate
*Rudbeckia* 45 GY M1	83.772	3.382	8.26	17.680	17	intermediate	intermediate

**Table 2:** Categorization of species based on APTI values.

Ganguly and Mukherjee [35]		Singh et al. [36]	
<1	very sensitive	<14	sensitive
1–16	sensitive	15–19	intermediate
17–29	intermediate	20–24	moderately tolerant
30–100	tolerant	>24	tolerant

## Data Availability

Not applicable.
